# Comparative Prognostic Utility of Indexes of Microvascular Function Alone or in Combination in Patients With an Acute ST-Segment–Elevation Myocardial Infarction

**DOI:** 10.1161/CIRCULATIONAHA.116.022603

**Published:** 2016-12-05

**Authors:** David Carrick, Caroline Haig, Nadeem Ahmed, Jaclyn Carberry, Vannesa Teng Yue May, Margaret McEntegart, Mark C. Petrie, Hany Eteiba, Mitchell Lindsay, Stuart Hood, Stuart Watkins, Andrew Davie, Ahmed Mahrous, Ify Mordi, Ian Ford, Aleksandra Radjenovic, Keith G. Oldroyd, Colin Berry

**Affiliations:** From BHF Glasgow Cardiovascular Research Centre, Institute of Cardiovascular and Medical Sciences (D.C., N.A., J.C., V.T.Y.M., M.M., M.C.P., I.M., A.R., K.G.O., C.B.), and Robertson Centre for Biostatistics (C.H., I.F.), University of Glasgow, Glasgow, UK; and West of Scotland Heart and Lung Centre, Golden Jubilee National Hospital, Clydebank, UK (D.C., N.A., J.C., V.T.Y.M., M.M., M.C.P., H.E., M.L., S.H.., S.W., A.D., A.M., I.M., K.G.O., C.B.).

**Keywords:** magnetic resonance imaging, microcirculation, myocardial infarction, prognosis

## Abstract

**Background:**

Primary percutaneous coronary intervention is frequently successful at restoring coronary artery blood flow in patients with acute ST-segment–elevation myocardial infarction; however, failed myocardial reperfusion commonly passes undetected in up to half of these patients. The index of microvascular resistance (IMR) is a novel invasive measure of coronary microvascular function. We aimed to investigate the pathological and prognostic significance of an IMR>40, alone or in combination with a coronary flow reserve (CFR≤2.0), in the culprit artery after emergency percutaneous coronary intervention for acute ST-segment–elevation myocardial infarction.

**Methods:**

Patients with acute ST-segment–elevation myocardial infarction were prospectively enrolled during emergency percutaneous coronary intervention and categorized according to IMR (≤40 or >40) and CFR (≤2.0 or >2.0). Cardiac magnetic resonance imaging was acquired 2 days and 6 months after myocardial infarction. All-cause death or first heart failure hospitalization was a prespecified outcome (median follow-up, 845 days).

**Results:**

IMR and CFR were measured in the culprit artery at the end of percutaneous coronary intervention in 283 patients with ST-segment–elevation myocardial infarction (mean±SD age, 60±12 years; 73% male). The median IMR and CFR were 25 (interquartile range, 15–48) and 1.6 (interquartile range, 1.1–2.1), respectively. An IMR>40 was a multivariable associate of myocardial hemorrhage (odds ratio, 2.10; 95% confidence interval, 1.03–4.27; *P*=0.042). An IMR>40 was closely associated with microvascular obstruction. Symptom-to-reperfusion time, TIMI (Thrombolysis in Myocardial Infarction) blush grade, and no (≤30%) ST-segment resolution were not associated with these pathologies. An IMR>40 was a multivariable associate of the changes in left ventricular ejection fraction (coefficient, −2.12; 95% confidence interval, −4.02 to −0.23; *P*=0.028) and left ventricular end-diastolic volume (coefficient, 7.85; 95% confidence interval, 0.41–15.29; *P*=0.039) at 6 months independently of infarct size. An IMR>40 (odds ratio, 4.36; 95% confidence interval, 2.10–9.06; *P*<0.001) was a multivariable associate of all-cause death or heart failure. Compared with an IMR>40, the combination of IMR>40 and CFR≤2.0 did not have incremental prognostic value.

**Conclusions:**

An IMR>40 is a multivariable associate of left ventricular and clinical outcomes after ST-segment–elevation myocardial infarction independently of the infarction size. Compared with standard clinical measures of the efficacy of myocardial reperfusion, including the ischemic time, ST-segment elevation, angiographic blush grade, and CFR, IMR has superior clinical value for risk stratification and may be considered a reference test for failed myocardial reperfusion.

**Clinical Trial Registration:**

URL: https//www.clinicaltrials.gov. Unique identifier: NCT02072850.

## Abstract 2

Supplemental Digital Content is available in the text.

Despite the success of emergency percutaneous coronary intervention (PCI) in restoring coronary blood flow in patients with acute ST-segment–elevation myocardial infarction (STEMI), a failure of myocardial reperfusion, which manifests initially as microvascular obstruction and then subsequently as myocardial hemorrhage, affects approximately half of patients with acute STEMI.^1,2^ Microvascular pathology (specifically, microvascular obstruction and myocardial hemorrhage) revealed by cardiac magnetic resonance (CMR) is prognostically important^3–5^; however, CMR is neither feasible acutely nor routinely recommended. Established tests for failed reperfusion such as the surface ECG, a test focused on ST-segment resolution and performed 60 to 90 minutes after reperfusion,^6^ and the angiographic tissue myocardial perfusion grade^7,8^ lack sensitivity and reproducibility in routine practice.^9^ Failed myocardial reperfusion passes undetected in up to half of patients after acute STEMI.^3,4^

Invasive assessment of microcirculatory function at the end of emergency PCI before the patient is transferred to the ward presents an opportunity to identify STEMI patients with failed myocardial reperfusion with greater accuracy than the angiogram or the ECG. The index of microvascular resistance (IMR) is independently associated with left ventricular (LV) function^10^ and infarct pathology,^11,12^ and in a recent study, an IMR>40 was a multivariable associate of mortality after STEMI.^13^ Coronary flow reserve (CFR) reflects epicardial and microvascular vasodilator capacity.^14^ CFR is associated with composite cardiovascular outcomes, including revascularization, in patients with stable coronary disease^15^ and after acute STEMI.^16^ We have recently shown that IMR is more closely associated with severe microvascular pathology, LV remodeling, and health outcome than either the angiogram or CFR,^17^ but whether the combination of IMR and CFR adds prognostic value is uncertain.

Different IMR cutoffs have been proposed,^10–13^ but only an IMR>40 is associated with mortality.^13^ The combination of an increased IMR and reduced CFR has been associated with enhanced detection of microvascular obstruction^18^ and viability and prognosis.^16^ However, in that study, only 10 major adverse cardiac and cerebrovascular events occurred, of which 5 were revascularizations. Changes in IMR and CFR within 24 hours after reperfusion have been associated with LV ejection fraction (LVEF).^19,20^ However, prior studies are limited by sample size (n=27–45 subjects),^10,20–22^ short follow-up (3–6 months),^10,18,20–22^ lack of association with spontaneous hard outcomes,^16^ and differences in cutoffs,^12,23^ supporting the case for definitive research.

Building on prior literature, we hypothesized that in patients with an acute STEMI, an IMR>40 would be more closely associated with infarct pathology and clinical outcomes than established angiographic and ECG measures of myocardial reperfusion and that, compared with IMR alone, the combination of an IMR>40 and a CFR≤2.0 might be more closely associated with infarct pathologies and prognosis. We measured IMR and CFR simultaneously in the culprit coronary artery immediately after emergency PCI in a large, unselected population of patients with acute STEMI.

## Methods

### Study Population and STEMI Management

We performed a prospective cohort study in a regional cardiac center between July 14, 2011, and November 22, 2012. Two hundred eighty-eight patients with STEMI were enrolled by 13 cardiologists. The patients provided written informed consent to undergo a diagnostic guidewire-based assessment after reperfusion and then CMR 2 days and 6 months later, as well as follow-up for health outcomes in the longer term.

Patients were eligible if they had an indication for primary PCI or thrombolysis for acute STEMI.^24,25^ Exclusion criteria included standard contraindications to CMR, for example, a pacemaker. The study was approved by the National Research Ethics Service (reference 10-S0703-28). Acute STEMI management (Methods in the online-only Data Supplement) followed contemporary guidelines.^24,25^ The ClinicalTrials.gov identifier is NCT02072850.

### Measurement of CFR and IMR in the Culprit Coronary Artery at the End of PCI

We adopted a thermodilution technique rather than Doppler because we wished to implement a method that is most transferable to routine clinical practice. In our experience, the Doppler measurements can be more time-consuming, require considerable experience, and may be less reproducible,^14^ and the guidewire is typically more expensive.

A coronary pressure- and temperature-sensitive guide wire (St. Jude Medical, St. Paul, MN) was used to measure IMR and CFR in the culprit coronary artery at the end of primary or rescue PCI. The guidewire was calibrated outside the body, equalized with aortic pressure at the ostium of the guide catheter. and then advanced to the distal third of the culprit artery. This thermodilution method is based on the following basic relationship: flow=volume/mean transit time. CFR is defined as the ratio of peak hyperemic to resting flow (CFR=flow at hyperemia/flow at rest). Flow is the ratio of the volume (V) divided by the mean transit time (Tmn). Thus, CFR can be expressed as follows: CFR=(V/Tmn) at hyperemia/(V/Tmn) at rest. Assuming that the epicardial volume remains unchanged, CFR can be calculated as follows: CFR=Tmn at rest/Tmn at hyperemia. CFR and IMR are distinct physiological parameters. CFR reflects epicardial and microcirculatory function. In contrast, IMR is a direct invasive measure of microvascular resistance. IMR is defined as the distal coronary pressure multiplied by the mean transit time of a 3-mL bolus of saline at room temperature during maximal coronary hyperemia measured simultaneously (mm Hg·s or units).^10–12^

Hyperemia was induced by 140 μg·kg^−1^·min^−1^ of intravenous adenosine preceded by a 2-mL intracoronary bolus of 200 µg nitrate. The mean aortic and distal coronary pressures were recorded during maximal hyperemia. We have previously found IMR to be highly repeatable when assessed by duplicate measurements 5 minutes apart in 12 consecutive patients with STEMI at the end of PCI.^12^

On the basis of prior literature, we prespecified and examined an IMR>40 and the following classifications: (1) IMR≤40 and CFR>2.0, (2) IMR>40 and CFR>2.0, (3) IMR≤40 and CFR≤2.0, and (4) IMR>40 and CFR≤2.0.

### CMR Imaging

**Figure 1.**
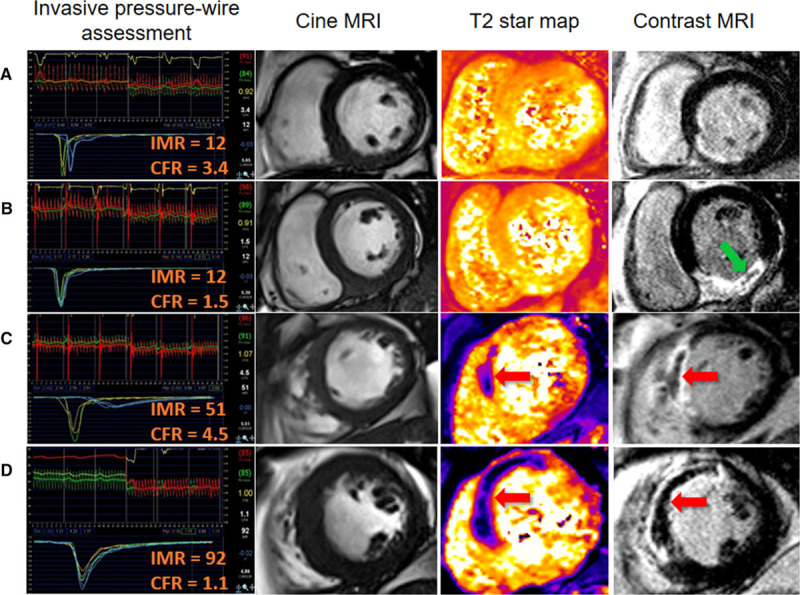
**Four patients with acute ST-segment–elevation myocardial infarctiontreated by primary percutaneous coronary intervention (PCI).** Each patient had index of microvascular resistance (IMR) and coronary flow reserve (CFR) measured in the culprit coronary artery at the end of the procedure. The patients reflect the following categories: IMR≤40 and CFR>2.0; IMR≤40 and CFR≤2.0; IMR>40 and CFR>2.0; and IMR>40 and CFR≤2.0. The patients were treated with similar antithrombotic therapy, including aspirin, clopidogrel, heparin, and intravenous glycoprotein IIb/IIIa inhibitor therapy with tirofiban. Each patient had normal TIMI (Thrombolysis in Myocardial Infarction) grade 3 flow at the end of PCI. Cardiac magnetic resonance imaging (MRI) was performed for each patient 2 days later. **A**, A patient with a normal IMR and a normal CFR. Invasive assessment of microvascular function in the culprit coronary artery at the end of primary PCI indicated that microcirculatory function was preserved. Cardiac magnetic resonance (CMR) subsequently revealed nontransmural late gadolinium enhancement consistent with salvaged myocardium. There was no evidence of myocardial hemorrhage (**middle right**) or microvascular obstruction (**right**). **B**, A patient with a normal IMR and a low CFR. Late gadolinium contrast CMR revealed transmural inferior myocardial infarction with a small central zone of hypointense microvascular obstruction (**arrow**, **right**). T2*-CMR excluded myocardial hemorrhage within the infarct core (**middle right**). **C**, A patient with a high IMR and a normal CFR. Late gadolinium contrast-enhanced CMR revealed transmural anteroseptal myocardial infarction complicated by microvascular obstruction (**arrow, right**). T2*-CMR (**arrow, middle right**) revealed myocardial hemorrhage within the infarct core, and microvascular obstruction spatially corresponded with the myocardial hemorrhage. **D**, A patient with a high IMR and a low CFR. Invasive guidewire-based physiological testing at the end of primary PCI revealed severe microvascular dysfunction. Transmural myocardial infarction and microvascular obstruction are present, in association with abundant myocardial hemorrhage (**arrow**, **middle right**).

We used CMR to provide reference data on LV function, pathology, and surrogate outcomes independently of the invasive tests (Figure 1). CMR was performed on a Siemens MAGNETOM Avanto (Erlangen, Germany) 1.5-T scanner with a 12-element phased-array cardiac surface coil.^26^ The imaging protocol^5,27^ (Methods in the online-only Data Supplement) included cine magnetic resonance imaging with steady-state free precession, T2 mapping,^28,29^ T2* mapping, and delayed-enhancement phase-sensitive inversion-recovery pulse sequences.^30^ The scan acquisitions were spatially coregistered and included different slice orientations to enhance diagnostic confidence.

### Imaging Analyses

The CMR analyses are described in detail in Methods in the online-only Data Supplement.

### Infarct Definition and Size

The presence of acute infarction was established on the basis of abnormalities in cine wall motion, rest first-pass myocardial perfusion, and delayed-enhancement imaging in 2 imaging planes. The myocardial mass of late gadolinium (grams) was quantified with computer-assisted planimetry, and the territory of infarction was delineated with the use of a signal intensity threshold of >5 SD above a remote reference region and expressed as a percentage of total LV mass.^31^

### Microvascular Obstruction

Microvascular obstruction was defined as a dark zone on early gadolinium enhancement imaging 1, 3, 5, and 7 minutes after contrast injection that remained present within an area of late gadolinium enhancement at 15 minutes.

### Myocardial Edema

The extent of myocardial edema was defined as LV myocardium with pixel values (T2) >2 SD from remote myocardium.^28,29,32–35^

### Myocardial Salvage

Myocardial salvage was calculated by subtracting the percent infarct size from percent area at risk, as reflected by the extent of edema.^12,32,35^ The myocardial salvage index was calculated by dividing the myocardial salvage area by the initial area at risk.

### LV Remodeling

An increase in LV volume at 6 months from baseline was taken to reflect LV remodeling.^27,35,36^ Adverse remodeling was defined as an increase in LV end-diastolic volume (LVEDV) ≥20% at 6 months from baseline.^27^

### Myocardial Hemorrhage

On the T2* CMR maps, a region of reduced signal intensity within the infarcted area with a T2* value of <20 milliseconds^4,37–40^ was considered to confirm the presence of myocardial hemorrhage.

### Electrocardiography

A 12-lead ECG was obtained before coronary reperfusion and 60 minutes afterward. The extent of ST-segment resolution on the ECG assessed 60 minutes after reperfusion compared with the baseline ECG before reperfusion^41^ was expressed as complete (≥70%), incomplete (30%–<70%), or none (≤30%).

### Coronary Angiogram Acquisition and Analyses

Coronary angiograms were acquired during usual care with cardiac catheter laboratory x-ray (Innova, GE Healthcare) and information technology equipment (Centricity, GE Healthcare). The angiograms were analyzed by trained observers (J.C., V.T.Y.M) who were blinded to all other clinical and MRI data. The TIMI (Thrombolysis in Myocardial Infarction) coronary flow grade^42^ and frame count^43^ were assessed at initial angiography and at the end of the procedure. TIMI myocardial perfusion grade^44^ was assessed at the end of the procedure (Methods in the online-only Data Supplement).

### Laboratory Analyses

The acquisition of blood samples for biochemical and hematologic analyses is described in Methods in the online-only Data Supplement.

### Prespecified Health Outcomes

We prespecified adverse health outcomes that are pathophysiologically linked with STEMI.^45,46^ The primary composite outcome was all-cause death or first heart failure event after the initial hospitalization (Methods in the online-only Data Supplement).

### Statistical Analyses

The sample size calculation and statistical methods are described in the Methods in the online-only Data Supplement. Random-effects models were used to compute interrater and intrarater reliability measures (intraclass correlation coefficient) for the reliability of angiographic measures of myocardial reperfusion measured independently by 2 observers in 20 randomly selected patients from the cohort (Results in the online-only Data Supplement). All *P* values are 2-sided, and value of *P* >0.05 indicates the absence of a statistically significant effect. Statistical analyses were performed with R version 2.15.1, SAS version 9.3, or higher versions of these programs.

## Results

### Patient Characteristics and IMR and CFR Measured Acutely in the Culprit Coronary Artery After Reperfusion

**Figure 2.**
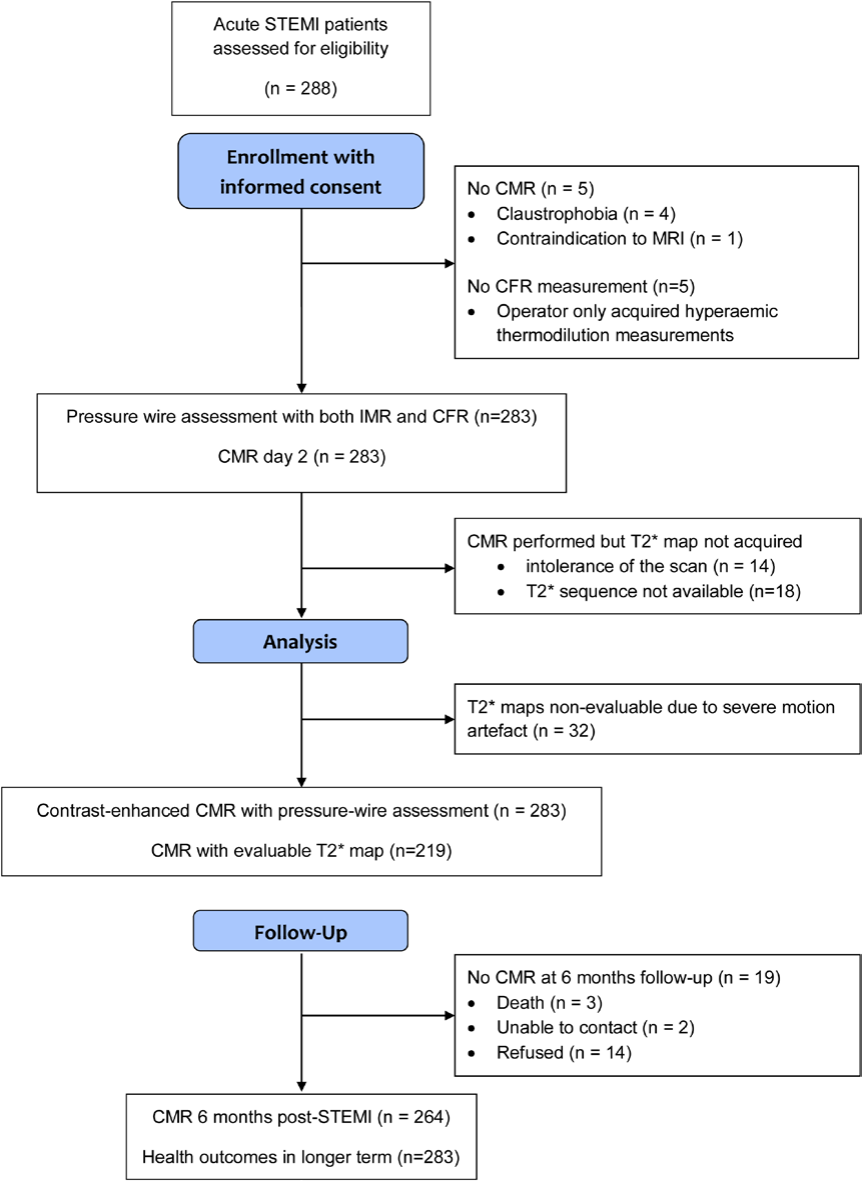
**CONSORT (*Consolidated* Standards of Reporting Trials) flow diagram of the cohort study.** CFR indicates coronary flow reserve; CMR, cardiac magnetic resonance; IMR, index of microvascular resistance; and STEMI, ST-segment–elevation myocardial infarction.

A total of 283 patients with STEMI had IMR and CFR measured in the culprit coronary artery at the end of emergency PCI (Table 1 and Figure 2). The median IMR and CFR were 25 (interquartile range, 15–48) and 1.6 (interquartile range, 1.1–2.1), respectively. A CFR≤2.0, an IMR>40, or both occurred in 210 (74%), 79 (28%) (Table 1), and 65 (23%) patients, respectively (Table I in the online-only Data Supplement).

**Table 1.**
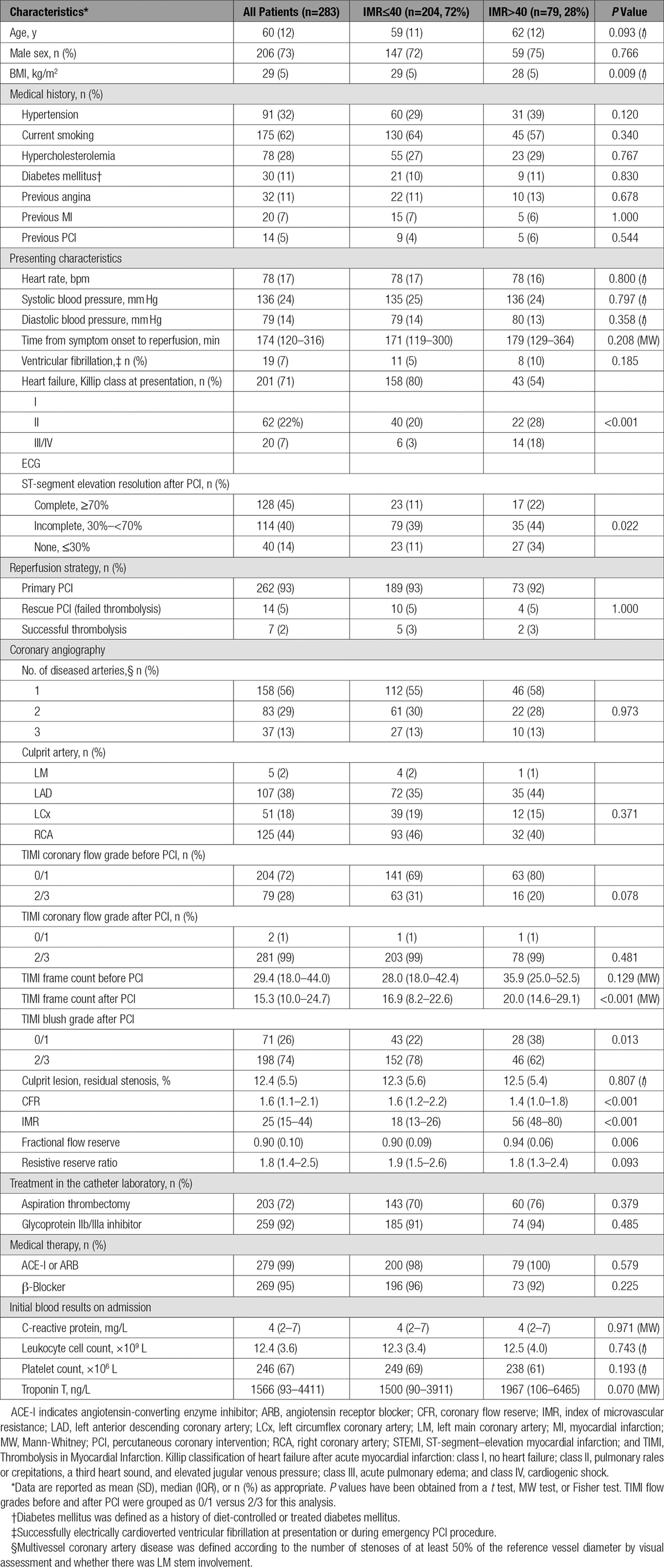
Clinical and Angiographic Characteristics of 283 Patients With STEMI Categorized According to an IMR≤40 or >40 Measured in the Culprit Coronary Artery at the End of PCI

### CMR Findings

CMR imaging occurred 2.1±1.8 days later, and 264 patients (93%) had follow-up CMR at 6 months (Table 2 and Figure 2). Case examples are shown in Figure 1. Myocardial hemorrhage and microvascular obstruction occurred in 89 (42%) and 114 (54%) patients, respectively. An IMR>40 (Table 2) and the combination of an IMR>40 and a CFR≤2.0 (Table II in the online-only Data Supplement) were associated with LVEF and infarct pathology 2 days after MI and LVEF at 6 months.

**Table 2.**
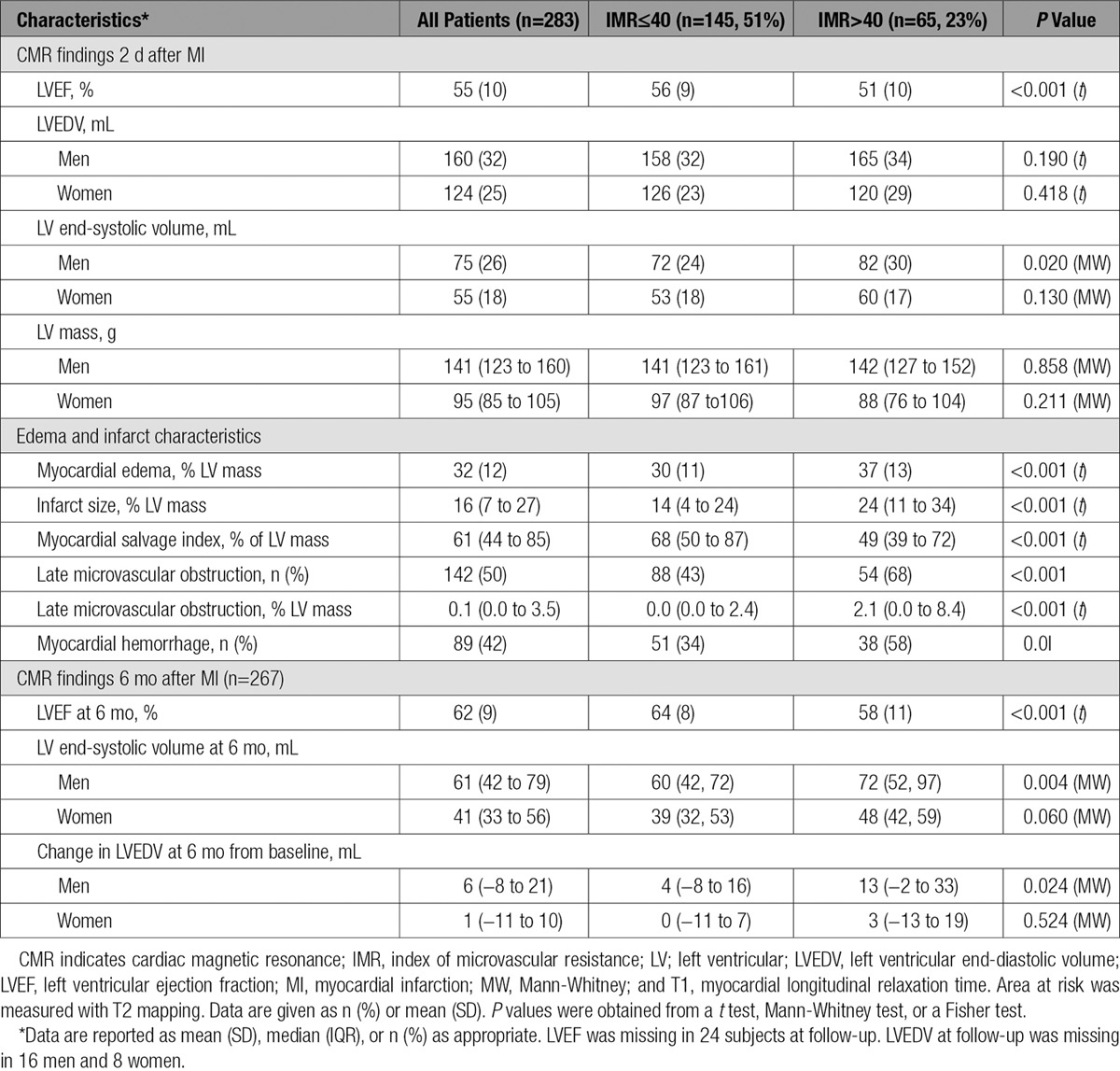
CMR Findings at 2 Days and 6 Months After Reperfusion in 283 Patients With STEMI Categorized According to an IMR ≤40 or >40 in the Territory of the Culprit Artery at the End of Emergency PCI

### Multivariable Associations for an IMR>40 With Microvascular Infarct Pathology Revealed by CMR

#### Myocardial Hemorrhage

In a binary logistic regression model with baseline characteristics, an IMR>40 was a multivariable associate of myocardial hemorrhage (odds ratio [OR], 2.86; 95% confidence interval [CI], 1.52–5.39; *P*=0.001; Table 3), whereas symptom-to-reperfusion time, TIMI blush grade, and no ST-segment resolution were not.

**Table 3.**
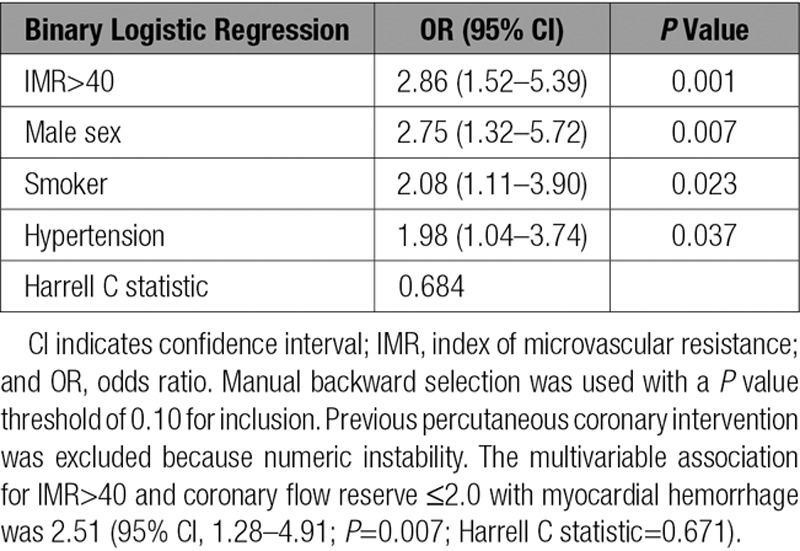
Multivariable Associations Between Clinical Characteristics, IMR>40 at the End of Emergency PCI, and the Occurrence of Myocardial Hemorrhage 2 Days Later (n=200) in Patients With Acute STEMI

#### Microvascular Obstruction

An IMR>40 was a multivariable associate of microvascular obstruction (OR, 2.82; 95% CI, 1.62–4.93; *P*<0.001; Table III in the online-only Data Supplement). Symptom-to-reperfusion time, TIMI blush grade, and no ST-segment resolution were not multivariable associates of microvascular obstruction.

#### Microvascular Infarct Pathologies and Invasive Microvascular Parameters in Combination

The combination of IMR>40 and CFR≤2.0 was a multivariable associate with microvascular obstruction (OR, 2.28; 95% CI, 1.16–4.46; *P*=0.016) but not with myocardial hemorrhage (*P*=0.104).

Compared with IMR>40 and CFR≤2.0 (reference group), the group with the combination of IMR≤40 and CFR≤2.0 was associated with a reduced odds of microvascular obstruction (OR, 0.19; 95% CI, 0.05–0.76; *P*=0.019) and myocardial hemorrhage (OR, 0.17; 95% CI, 0.03–0.92; *P*=0.040).

### Microvascular Dysfunction and Subsequent LV Outcomes

#### Changes in LVEDV

An IMR>40 was a univariable (regression coefficient, 11.43; 95% CI, 4.07–18.79; *P*=0.002) and a multivariable (regression coefficient, 7.85; 95% CI, 0.41–15.29; *P*=0.039) associate of the changes in LVEDV, including after adjustment for infarct size (n=264; Table 4).

**Table 4.**
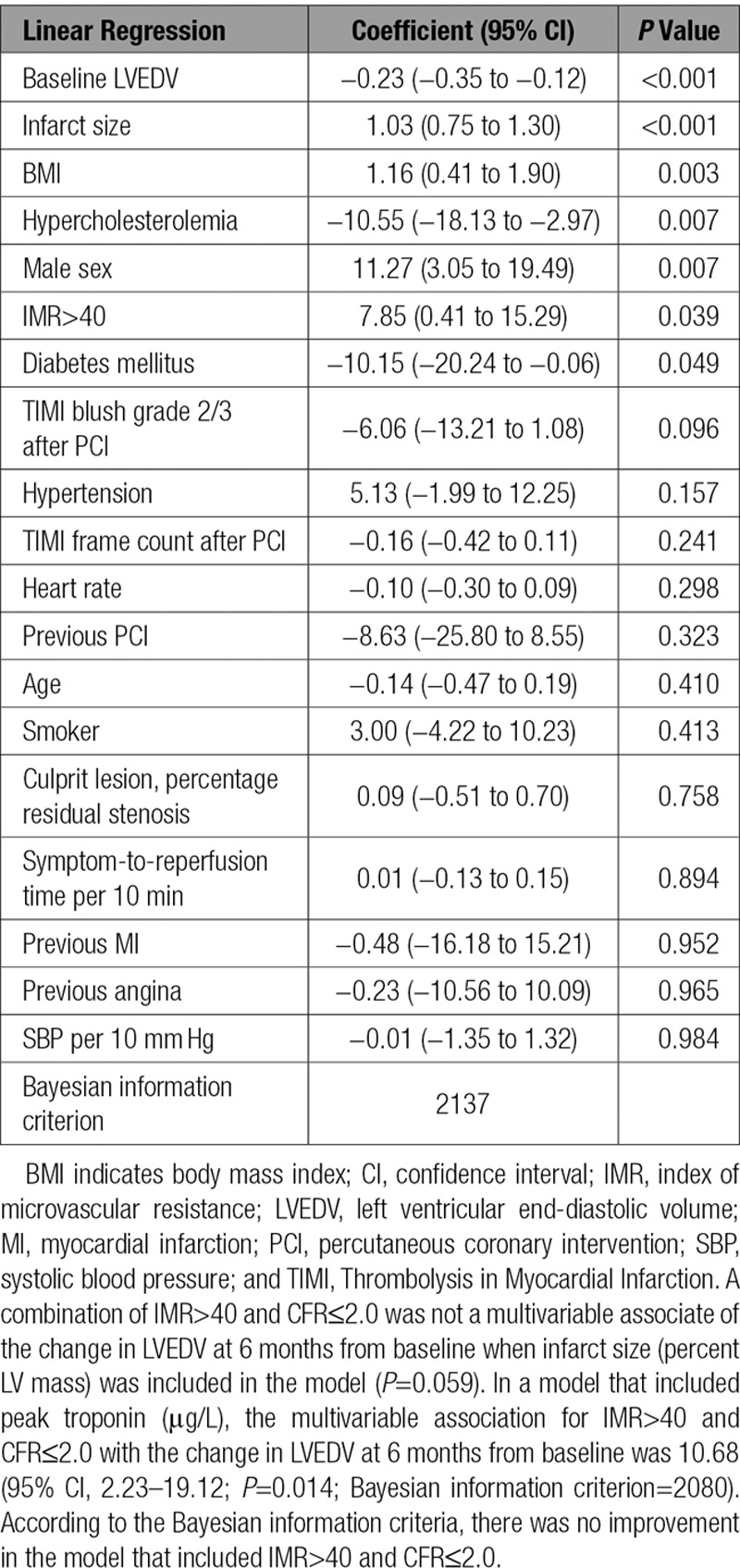
Multivariable Associations Between an IMR>40 and Changes in LVEDV at 6 Months From Baseline (n=264)

#### Changes in LVEF

An IMR>40 was a univariable (regression coefficient, −2.89; 95% CI, −4.89 to −0.91; *P*=0.004, with adjustment for baseline LVEF) and a multivariable (regression coefficient, −2.12; 95% CI, −4.02 to −0.23; *P*=0.028) associate of the changes in LVEF at 6 months from baseline, including after adjustment for infarct size, as reflected by troponin or contrast-enhanced MRI (n=264; Table IV in the online-only Data Supplement).

#### LV Outcomes and the Combination of IMR>40 and CFR≤2.0

Results for the multivariable models for IMR>40 combined with CFR≤2.0 were not improved compared with the model with IMR>40 (Tables IV and V in the online-only Data Supplement, footnote).

### Microvascular Dysfunction and Longer-Term Health Outcomes

All of the patients (n=283) had completed long-term follow-up data. The median duration of follow-up was of 845 days (range of postdischarge censor duration, 598–1098 days). Thirty patients (11%) died or experienced a first heart failure event during the index hospitalization or after discharge. These events included 5 cardiovascular deaths, 3 noncardiovascular deaths, and 22 episodes of heart failure (Killip class 3 or 4 heart failure [n=20] or defibrillator implantation [n=2]). Ten patients (3.5%) died or experienced a first heart failure hospitalization after discharge (Table V in the online-only Data Supplement).

IMR was a univariable associate of all-cause death or heart failure, whereas CFR was not (Table 5). Because of the number of events observed, 2 multivariable models were considered: 1 model with hypertension and smoking as covariates and 1 model with ST-segment resolution (none) and TIMI frame count (Table 5). In the model with smoking and hypertension, an IMR>40 (OR, 4.70; 95% CI, 2.10–10.53; *P*<0.001) was a multivariable associate of all-cause death or heart failure. In the model with ST-segment resolution (none) and TIMI frame count, an IMR>40 was also a multivariable associate with this outcome (OR, 4.42; 95% CI, 1.93–10.10; *P*<0.001). The combination of IMR>40 and CFR≤2.0 did not enhance the magnitude of the prognostic significance of IMR>40 (Table 5).

**Table 5.**
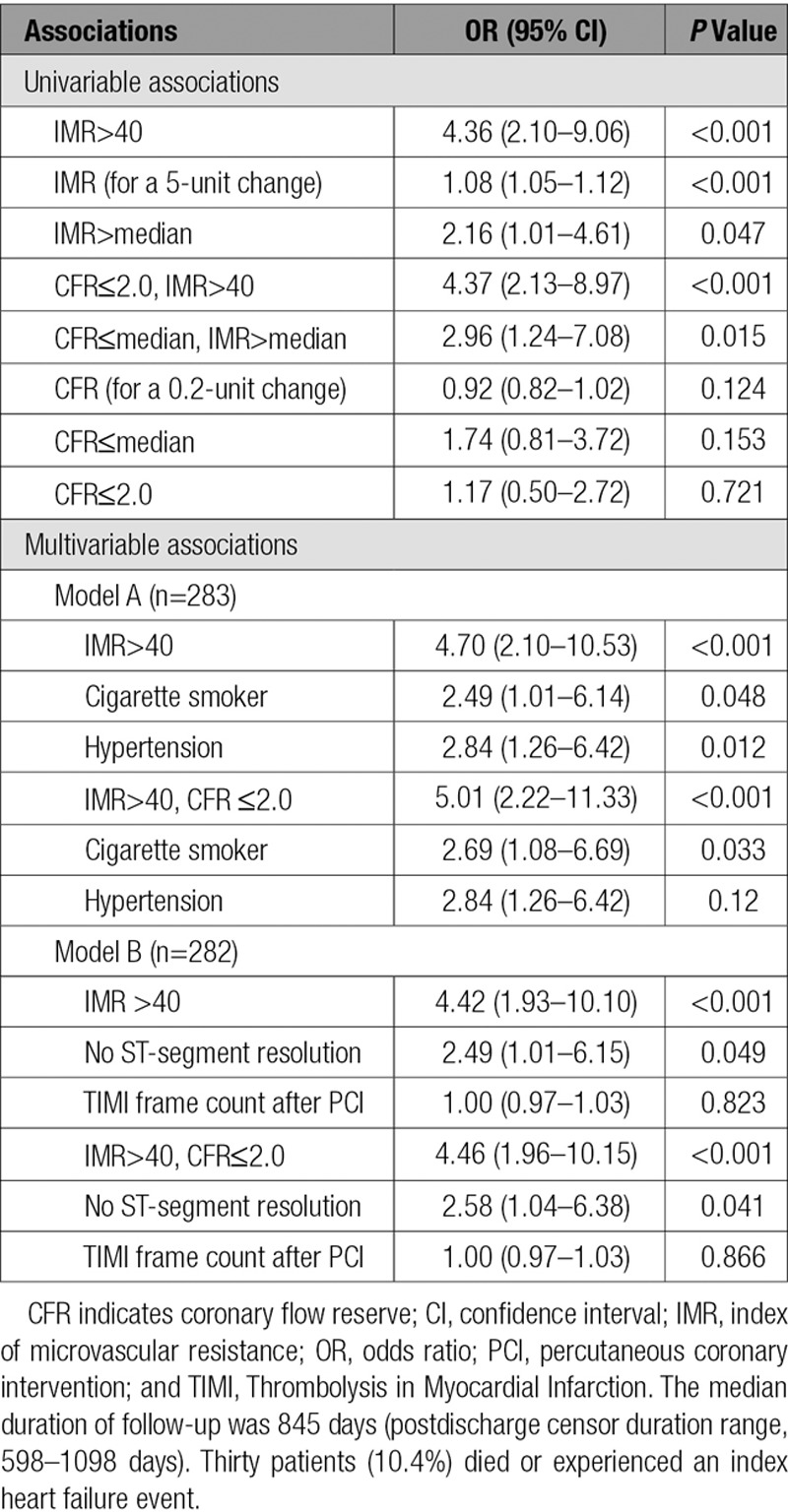
Relationships Between IMR and CFR and All-Cause Death or First Hospitalization for Heart Failure During or After the Index Hospitalization Obtained With Logistic Regression

### Fractional Flow Reserve and the Ratio of CFR to Fractional Flow Reserve

Fractional flow reserve measured in the culprit coronary artery was not associated with myocardial hemorrhage status (*P*=0.262), nor was it associated with LVEDV or LVEF at baseline or at follow-up. Fractional flow reserve was not associated with health outcomes. Similar results were observed for the ratio of CFR to fractional flow reserve, which reflects true CFR (Results in the online-only Data Supplement).

## Discussion

We have undertaken the largest prospective study of invasive tests of microvascular function, infarct pathology revealed by serial CMR, and spontaneous adverse health outcomes in patients with acute STEMI.

The main findings are the following: (1) Microvascular dysfunction at the end of emergency PCI, as classified by an IMR>40 (without CFR), was more consistently associated with infarct pathology and prognosis than symptom-to-reperfusion time or angiographic and ECG measures of reperfusion; (2) an IMR>40 was more closely associated with myocardial hemorrhage and microvascular obstruction than the combination of an IMR>40 and CFR≤2.0; (3) an IMR>40 was a multivariable associate of the changes in LVEF and LVEDV independently of infarct size; and (4) an IMR>40 identifies patients who have a 4-fold increase in all-cause death or heart failure, whereas CFR (or true CFR) alone was not associated with this outcome and the combination of IMR and CFR had no incremental prognostic value. These results refute our hypothesis that the combination of IMR with CFR would have superior prognostic value.

### Implications for Patient Management

Using IMR in patients with acute STEMI, the cardiologist can focus risk stratification with a simple index that has a single cutoff (IMR>40). This test of microvascular dysfunction provides incremental prognostic information over and above infarct size at an early time point before infarct size is disclosed by measurement of troponin or MRI. This result enhances the clinical relevance of measuring IMR in patients with acute STEMI. CFR, either alone or in combination with IMR, is not needed, and a more complicated combined approach with both measures is not necessary.

Our study adds to the literature on the invasive assessments of the efficacy of myocardial reperfusion in patients with acute STEMI.^11–13,20–22,47^ Fearon et al^13^ established that an IMR>40 was independently associated with all-cause mortality and heart failure; however, information on LV function and infarct pathology was not described, and the IMR threshold of 40 lacks validation against infarct pathology and LV outcomes. Our study includes new information with serial CMR. We have shown that an IMR>40 is independently associated with infarct pathology, changes in LV function and volume, and all-cause-death or heart failure. On the other hand, the prognostic significance of CFR was less than that of IMR, and CFR was not additive to IMR. CFR has greater hemodynamic dependence; it is subject to variations in resting flow, is not specific for the microvasculature, and has a narrower range of values.^14,48^

CFR reflects the functional (vasodilator) capacity of the coronary artery circulation,^48^ whereas IMR reflects microvascular resistance. Park et al^16^ undertook a prognostic study of IMR and CFR in 89 patients with acute STEMI. They found that the combination of an increased IMR and reduced CFR was associated with changes in LV wall motion score index at 3 months as revealed by echocardiography and major adverse cardiac and cerebrovascular events. The results of this study lend support to the theory that the combination of IMR and CFR might have additive prognostic value compared with either index alone. Compared with the study by Park et al,^16^ our study included a population that was 3 times larger, advanced cardiac imaging with MRI, independent analysis of spontaneous adverse cardiac events, and a composite outcome that did not include revascularization. Furthermore, another small study (n=40)^18^ in patients with acute STEMI showed that the combination of high IMR and low CFR enhanced the predictive accuracy of detecting microvascular obstruction compared with either index alone. The results from our study refute those of Park et al^16^ and Ahn et al^18^ and indicate that an IMR>40 is sufficient for prognostication.

In the acute clinical setting, failed myocardial reperfusion, as reflected by microvascular obstruction and myocardial hemorrhage, occurs in about half of all patients with STEMI and commonly passes undetected acutely. Microvascular obstruction is potentially reversible,^4^ but without successful myocardial reperfusion, severe vascular damage progresses to irreversible myocardial hemorrhage in 40% of all patients.^3–5^ When CMR is performed days later, it is too late for early intervention to prevent or treat severe microvascular damage, and CMR has limited availability in routine practice.

An IMR>40 was consistently associated with infarct pathology, changes in LV function and volumes independently of infarct size, and all-cause death or heart failure compared with other standard measures of reperfusion injury, including TIMI frame count, TIMI myocardial perfusion grade, and ST-segment resolution.^24,49^ In our population, a minority of patients (14%) had no evidence of ST-segment resolution 60 minutes after reperfusion, yet microvascular obstruction and myocardial hemorrhage occurred in 50% and 42% of patients, respectively. TIMI myocardial perfusion grade was not associated with clinical outcomes (Table 5) and is difficult to reliably measure in clinical practice. Reliable measurement of failed reperfusion at the end of the PCI procedure is therefore a difficult clinical conundrum, not least because coronary reperfusion is successfully achieved in the majority of all patients.

Our results have important clinical implications. Failed myocardial reperfusion in patients with acute STEMI is common, is associated with adverse outcome, and often goes unnoticed, largely because current assessment methods lack sensitivity and routine CMR, usually performed days after the acute event, is often not practical or cost-efficient. Immediate detection of failed myocardial reperfusion becomes feasible with IMR, is safe,^50^ and allows direct stratification of the highest-risk patients at the time of emergency reperfusion, when early therapeutic interventions may yield the greatest clinical benefit. Conversely, the possibility remains that an IMR>40 may represent an unmodifiable marker of elevated risk.

### Implications for Therapy and Clinical Trials

Further research is warranted to investigate preventive or therapeutic interventions in patients stratified by IMR to assess whether IMR-guided strategies might improve prognosis compared with standard care.

Our results provide evidence both for and against IMR as identifying modifiable risk (hence a target for treatment) as opposed to being only an unmodifiable marker of elevated risk (and hence not a target for treatment). The modifiable associations include myocardial salvage index, microvascular obstruction, and myocardial hemorrhage (all of which are linked to the pathophysiology of LV remodeling), and nonmodifiable associations (eg, body mass index, Killip class at presentation, area at risk [myocardial edema] which are essentially markers for increased myocardial mass at risk). Although IMR might offer an opportunity to guide therapy, it may mostly reflect a larger area at risk and thus be unmodifiable. Only an outcomes-based, randomized, controlled trial will decide the issue.

There is some evidence that IMR is responsive to the effects of treatments known to have favorable cardiovascular effects, including vasodilators^51^ and anti-ischemic^52^ therapies. During PCI, compared with a direct stenting approach without initial balloon angioplasty, a predilatation step to disrupt and modify the plaque before stenting is associated with a higher IMR at the end of the PCI procedure.^53^ In the setting of acute STEMI, a randomized trial of initial antiplatelet therapy in 76 patients undergoing primary PCI disclosed that, compared with an oral loading dose of 600 mg clopidogrel, an oral loading dose of 180 mg ticagrelor was associated with a lower IMR at the end of the procedure (22.2±18.0 versus 34.4±18.8 U; *P*=0.005).^54^ In other randomized, controlled trials in acute MI, IMR is being used to assess the comparative efficacy of antiplatelet therapies^55^ (NCT0273334), vasodilator therapy,^56^ and low-dose intracoronary thrombolysis (T-TIME [A Trial of Low-Dose Adjunctive alteplase During Primary PCI]; NCT02257294).

### Sample Size Calculation and Clinical Trials

In addition to the study design, estimated treatment effect, and power, the key factor that will influence the sample size in a clinical trial in which IMR is used as measure of treatment effect is the variance in IMR for the population studied. T-TIME is a randomized, placebo-controlled trial of 2 reduced doses of alteplase (10 and 20 mg) administered directly into the culprit coronary artery after reperfusion but before stent implantation. In that trial, we have estimated that if the median IMR is 33.9 (SD, 25.2) and the IMR values are 27.2 and 20.5 in the 10- and 20-mg dose groups, respectively, then 80 subjects per group would be needed. This calculation is based on an average difference in IMR between treatment and placebo of 10, assuming that there is a linear trend with dose. If the average difference in IMR between treatment and placebo is 13, then 48 subjects per group would be needed.

### Limitations

We performed a single-center, natural-history study. The median IMR in our population was 25, which is comparable to previous IMR values in some^12,23^ but not all^11,13^ cohorts of patients with STEMI. IMR is associated with infarct size^11^ and potentially the duration of ischemia. The ischemic time in our population was relatively short (Table 1), which potentially explains IMR distribution in our population. There was a comparatively lower proportion of patients with an anterior STEMI in our cohort (37% of patients) compared with, for example, 49% of cases in the study by McGeoch et al^11^ (median IMR, 35) and 55% of cases in the study by Fearon et al^13^ (median IMR, 31). These studies involved fewer patients, and enrollment may have been more selective. IMR measurement involves a diagnostic guidewire and use of intravenous adenosine and may prolong the procedure by ≈5 minutes. In 2013, the US Food and Drug Administration issued a safety announcement on the risk of MI and death in patients receiving Adenoscan (adenosine) for stress testing. However, a subsequent prospective, multicenter study has shown that intravenous adenosine when administered briefly for invasive physiology testing is safe and well tolerated in patients with acute or recent MI.^50^ IMR was measured routinely in our catheter laboratories, with measurements obtained by all of the cardiologists (n=13) without complication and in the setting of routine emergency care.

Most of the adverse events occurred initially during the index hospitalization. The limited number of adverse events constrained the statistical power of the multivariable models of adverse health outcomes. The study population included 21 patients initially treated with thrombolysis, and 14 of these patients had rescue PCI. The main results of our study were unchanged when these patients were removed (data not shown). The limited number of adverse events constrained the number of variables and related statistical power in the prognostic models. Our analysis does not permit inference on causality, and further studies are warranted.

### Conclusions

Compared with the angiographic and ECG measures of reperfusion, the combination of IMR>40 and CFR≤2.0, and CFR alone, an IMR>40 is consistently and strongly associated with microvascular pathology, changes in LV function and volumes, and all-cause death and heart failure in the longer term. Our results validate previous investigations and support further research into IMR-based therapeutic strategies.

## Acknowledgments

The authors thank the patients who participated in this study and the staff in the Cardiology and Radiology departments. The authors thank Peter Weale and Patrick Revell (Siemens Healthcare, UK).

## Sources of Funding

This work was supported by the British Heart Foundation Center of Research Excellence Award (RE/13/5/30177), the British Heart Foundation Project grant PG/11/2/28474, the National Health Service, and the Chief Scientist Office. Dr Berry was supported by a Senior Fellowship from the Scottish Funding Council. Dr Welsh is supported by British Heart Foundation Fellowship FS/12/62/29889.

## Disclosures

On the basis of institutional agreements with the University of Glasgow, Siemens Healthcare has provided work-in-progress imaging methods, and Dr Berry has acted as a consultant to St. Jude Medical. Dr Oldroyd has acted as consultant to St. Jude Medical and Volcano Corporation. These companies had no involvement in the current research or the manuscript. The other authors report no conflicts.

## Supplementary Material

## Clinical Perspective

### What Is New?

- The index of microvascular resistance (IMR) and coronary flow reserve were routinely measured in the culprit coronary artery of a reasonably large cohort of patients with acute ST-segment–elevation myocardial infarction treated by emergency percutaneous coronary intervention.
- Compared with ischemic time and angiographic and electrocardiographic measures of reperfusion, an IMR>40 was more consistently and strongly associated with microvascular pathology, changes in left ventricular function and volumes, and all-cause death and heart failure in the longer term.
- Compared with an IMR>40, the combination of IMR>40 and coronary flow reserve ≤2.0 did not have additional prognostic value.

### What Are the Clinical Implications?

- Despite the routine success of primary percutaneous coronary intervention, failed myocardial reperfusion is common and usually passes undetected.
- IMR has emerging clinical utility as a routine test for the efficacy of myocardial reperfusion in invasively managed patients with acute ST-segment–elevation myocardial infarction.
- An IMR>40 represents a prognostically validated reference test for failed myocardial reperfusion at the end of primary percutaneous coronary intervention.
- Our results confirm previous investigations and support further research into IMR-based therapeutic strategies in patients with acute ST-segment–elevation myocardial infarction.
